# Mr. Robert Grant

**Published:** 1910-07-23

**Authors:** 


The death is announced of Mr. Robert Grant, C.B., M.B., M.A., Inspector-General of Hospitals and Fleets, R.N. (retired), at the age of sixty-eight. He studied at the University of Aberdeen, where he graduated M.B., B.S., in 1866. In 1868 he joined the Navy, and served in the Zulu and Kaffir Wars of 1877-9, having charge of the hospital ship at Capetown during an outbreak of smallpox. For this he received a medal and promotion. He served in the Egyptian War of 1882, and gained the Khedive's Bronze Star. In 1888 he became Fleet-Surgeon, Deputy Inspector-General in 1897, and Inspector-General in 1901. He received the C.B. at King Edward's coronation.

